# Häusliche Pflegearrangements: Untersuchung der Inanspruchnahme von Unterstützungsleistungen im Zusammenhang mit dem sozioökonomischen Status

**DOI:** 10.1007/s00103-023-03684-6

**Published:** 2023-03-20

**Authors:** Nathalie Englert, Lara Peters, Mareike Przysucha, Marco Noelle, Manfred Hülsken-Giesler, Andreas Büscher

**Affiliations:** 1 Fakultät Wirtschafts- und Sozialwissenschaften, Hochschule Osnabrück, Caprivistr. 30a, 49076 Osnabrück, Deutschland; 2 Fachbereich Gesundheit, Fachhochschule Münster, Münster, Deutschland; 3 Fachbereich Humanwissenschaften, Institut für Gesundheitsforschung und Bildung, Universität Osnabrück, Osnabrück, Deutschland; 4 Fakultät für Gesundheit, Department für Pflegewissenschaft, Universität Witten/Herdecke, Witten, Deutschland

**Keywords:** Soziale Ungleichheit, Versorgungsangebote, Häusliche Pflege, Inanspruchnahme, Deutschland, Social inequality, Care services, Homecare, Service utilization, Germany

## Abstract

**Hintergrund:**

Nationale und internationale Befunde weisen auf den Einfluss sozioökonomischer Faktoren auf häusliche Pflegearrangements hin. Demografische und soziale Entwicklungen begründen die Annahme einer steigenden Zahl pflegebedürftiger Menschen aus ressourcenschwachen Gruppen und damit die zunehmende Bedeutung der Langzeitversorgung im häuslichen Umfeld.

**Ziel der Arbeit:**

Es wird untersucht, ob Einkommen und Bildung der pflegebedürftigen Menschen und ihrer Pflegepersonen mit der Inanspruchnahme von Unterstützungsleistungen bei der Gestaltung häuslicher Pflegearrangements in Zusammenhang stehen.

**Material und Methoden:**

Quantitative Sekundärdatenanalyse einer Befragung von Mitgliedern des Sozialverbands VdK. Die statistische Auswertung umfasst deskriptive Analysen sowie die Analyse von Zusammenhängen zwischen sozioökonomischen Merkmalen und Merkmalen des Pflegearrangements.

**Ergebnisse:**

Ein Zusammenhang von Einkommen und Versorgungsnutzung kann bei der Inanspruchnahme der sog. 24-Stunden-Pflege nachgewiesen werden, die häufiger bei hohem Einkommen zu finden ist. Weitere Einkommenseffekte zeigen sich beim Nutzungsumfang von Pflegediensten und Haushaltshilfen sowie bei wohnraumanpassenden Maßnahmen. Höhere Bildung geht mit einer gesteigerten Nutzung von Beratungsangeboten einher. Die Einschätzung der Pflegesituation fällt negativer aus, je niedriger das Einkommen ist.

**Diskussion:**

Die Ergebnisse weisen darauf hin, dass Phänomene sozialer Ungleichheit in der Pflege existieren und die Möglichkeiten zur Gestaltung häuslicher Pflege durch sozioökonomische Faktoren beeinflusst werden. Zugleich zeigt die Studie Herausforderungen bei der Auseinandersetzung mit sozialer Ungleichheit auf und gibt Orientierung für weitere Forschung, die angesichts gesellschaftlicher Trends an Bedeutung gewinnt.

**Zusatzmaterial online:**

Zusätzliche Informationen sind in der Online-Version dieses Artikels (10.1007/s00103-023-03684-6) enthalten.

## Abstract (en)

**Background:**

National and international findings indicate that homecare arrangements are influenced by socioeconomic factors. Demographic and social developments justify the assumption of an increasing number of people in need of care from resource-pour groups and therefore a continuously increasing importance of home-based long-term care.

**Objective:**

It is examined whether income and education of people in need of care and their caregivers are related to the use of support services in homecare arrangements.

**Material and methods:**

Quantitative secondary data analysis of a survey among members of the social association VdK (Sozialverband VdK). The statistical evaluation includes descriptive analyses as well as the investigation of correlations between socioeconomic characteristics and characteristics of the care arrangement.

**Results:**

A correlation between income and the use of support services exists regarding so-called 24-hour care, which is more common with high incomes. Other income effects can be seen in the extent to which care services and home help services are used and in measures to adapt the home environment. A higher level of education is related to an increased use of counselling services. The assessment of the care situation is more negative the lower the income.

**Conclusions:**

The results point out that phenomena of social inequality in care exist and that the options for shaping homecare are influenced by socioeconomic factors. The study also shows challenges in dealing with social inequality and provides orientation for further research, which is becoming increasingly important in view of current trends.

## Einleitung

Ungleichheiten in der pflegerischen Versorgung finden in Deutschland nur schleppend Eingang in die politische und wissenschaftliche Diskussion [1, 2]. Dies verwundert sowohl vor dem Hintergrund zahlreicher Erkenntnisse zu ungleicher medizinischer Versorgung (vgl. etwa [3]) als auch in Anbetracht der demografisch bedingten stetig zunehmenden Bedeutung pflegerischer Langzeitversorgung. Von Pflegebedürftigkeit im Sinne des Elften Buches Sozialgesetzbuch (SGB XI) sind in Deutschland derzeit 4,1 Mio. Menschen betroffen, 80 % davon leben zu Hause [4].

Die Pflege zu Hause entspricht nicht nur der präferierten Versorgungsform der allermeisten Menschen, sondern ist auch politisch mit dem Grundsatz „ambulant vor stationär“ ausdrücklich gewünscht. Ein häusliches Pflegearrangement umfasst „die Gesamtheit der pflegerischen Unterstützung in der häuslichen Umgebung eines pflegebedürftigen Menschen“ [5]. Dazu stehen verschiedene Leistungen und Unterstützungsmöglichkeiten zur Verfügung. Hauptleistungen der Pflegeversicherung sind die Sachleistung für die Unterstützung durch einen Pflegedienst sowie die Geldleistung für selbst beschaffte Pflegehilfen (Pflegegeld). Weitere Leistungsangebote bestehen in der Verhinderungspflege sowie Kurzzeit‑, Tages- und Nachtpflege in teilstationären Einrichtungen. Ein Entlastungsbetrag von monatlich 125 € kann zweckgebunden für Entlastungsangebote im Alltag eingesetzt werden, etwa Angebote zur Betreuung demenziell Erkrankter oder zur Unterstützung im Haushalt. Nicht von der Pflegeversicherung gedeckt ist die sogenannte 24-Stunden-Pflege, die an die Abnahme familialen Pflegepotentials anknüpft und zunehmend an Bedeutung gewinnt [6, 7], von den Betroffenen aber selbst finanziert werden muss. Zur Erleichterung und Verbesserung der Pflege bestehen darüber hinaus noch Ansprüche auf Pflegehilfsmittel sowie auf wohnumfeldverbessernde Maßnahmen. Weiterhin können Pflegebedürftige und Angehörige verschiedene Beratungsangebote nutzen bzw. haben Beratung zur Pflege bei ausschließlichem Pflegegeldbezug obligatorisch in Anspruch zu nehmen.

Aufgrund des Teilleistungscharakters der Pflegeversicherung ist die Inanspruchnahme dieser Angebote häufig mit nicht unerheblichen Zuzahlungen verbunden [8, 9] und erfordert zudem von den Betroffenen anspruchsvolle bürokratische Antragsleistungen. Dies begründet die Annahme, dass die Möglichkeiten zur Gestaltung häuslicher Pflege sozioökonomisch determiniert sind. Demografische und soziale Entwicklungen weisen zudem auf eine steigende Zahl pflegebedürftiger Menschen aus ressourcenschwachen Gruppen hin [10, 11], was die Dringlichkeit der Auseinandersetzung mit Fragen sozialer Ungleichheit in der häuslichen Pflege verdeutlicht.

Soziale Ungleichheit beschreibt die systematische und gesellschaftlich verankerten Strukturen folgende ungleiche Verteilung von Lebens- und Handlungschancen zwischen verschiedenen Gruppen einer Gesellschaft [12]. In modernen Gesellschaften gelten das Einkommen bzw. Vermögen, der Bildungsgrad sowie die berufliche Stellung als wichtigste Determinanten sozialer Ungleichheit und bilden die Grundlage zur Bestimmung des sozioökonomischen Status [13]. Auf internationaler Ebene setzt sich eine wachsende Anzahl von Studien mit Ungleichheit bei der Gestaltung von Pflegearrangements auseinander. Wenngleich die Evidenz noch inkonsistent ist, zeigen die Befunde, dass eine Verbindung zwischen dem sozioökonomischen Status und der Nutzung von Versorgungsangeboten besteht [14–18]. Insgesamt scheinen sozial determinierte Ungleichheiten bei der Gestaltung häuslicher Pflegearrangements zu entstehen, je höher in einem nationalen Pflegesystem der private Finanzierungsanteil bei der Inanspruchnahme von Versorgungsangeboten ist und je mehr die Pflege sowohl kulturell als auch pflegepolitisch in familiale Verantwortung gegeben wird [19–21]. Bisherige Befunde aus Deutschland weisen ebenfalls darauf hin, dass häusliche Pflegearrangements von sozioökonomischen Faktoren beeinflusst werden. Dabei wird in qualitativen Studien deutlich, dass Pflegekräfte deutliche Restriktionen für pflegebedürftige Personen aus einkommensschwachen Statusgruppen wahrnehmen [22, 23]. Quantitativ lässt sich eine ungleiche Verteilung der Versorgungsnutzung bislang nicht eindeutig abbilden [24–26].

Vor diesem Hintergrund wird in der vorliegenden Studie anhand von Sekundärdaten aus einer Befragung von Mitgliedern des Sozialverbandes VdK die Gestaltung häuslicher Pflegearrangements im Zusammenhang mit dem sozioökonomischen Status untersucht. Im Fokus steht die Frage, wie Einkommen und Bildung von pflegebedürftigen Menschen und ihren Pflegepersonen die Inanspruchnahme von Unterstützungsleistungen beeinflussen.

## Methoden

### Datengrundlage und Stichprobenkonstruktion

Die Datengrundlage bilden Ergebnisse einer Onlinebefragung unter Mitgliedern des Sozialverbandes VdK. Der VdK versteht sich als parteipolitisch und konfessionell unabhängige sozialpolitische Interessensvertretung seiner 2,1 Mio. Mitglieder [27]. Die Befragung wurde als Querschnittstudie von März bis Mai 2021 als Vollerhebung durchgeführt.

Mit unterschiedlichen Fragebögen wurden pflegebedürftige Personen, Personen mit Erfahrung als pflegende Angehörige sowie Personen, die über keine Erfahrung in der häuslichen Pflege verfügen, befragt [28]. Insgesamt haben sich 55.925 Personen an der Befragung beteiligt. Für die hier vorgestellte Sekundäranalyse wurden ausschließlich die Daten aus der Befragung von pflegebedürftigen Personen (*n* = 6589) sowie jener mit Pflegeerfahrung (im Folgenden als „Pflegepersonen“ bezeichnet; *n* = 27.351) genutzt, um Aussagen auf der Basis bestehender Versorgungsarrangements treffen zu können. In die Stichprobe einbezogen wurden ausschließlich Befragte, die pflegebedürftig im Sinne von § 14 SGB XI sind oder eine entsprechend pflegebedürftige Person pflegen.

Weiterhin wurden nur pflegebedürftige Personen ab 55 Jahren einbezogen. Hintergrund ist einerseits, dass dies die überwiegende Mehrheit pflegebedürftiger Menschen abbildet: Mehr als 80 % der Pflegebedürftigen sind älter als 55 Jahre [4]. Andererseits ist bei pflegebedürftigen Kindern, Jugendlichen und jungen Erwachsenen zu berücksichtigen, dass im Regelfall der sozioökonomische Status der Eltern die soziale Lage definiert, was eine gesonderte Betrachtung erfordert (Hinweise dazu finden sich etwa in [29]). Pflegepersonen wurden dagegen unabhängig von ihrem Alter einbezogen, sofern sie eine Person pflegen, die 55 Jahre oder älter ist.

### Sozioökonomische Merkmale

#### Einkommen.

Das Einkommen wurde durch Selbstauskunft als monatliches Netto-Einkommen des Haushaltes erfasst. Zu berücksichtigen waren alle Einkommensquellen des Haushaltes, neben Einkünften aus beruflicher Tätigkeit, Renten- oder Pensionszahlungen also auch Einkünfte aus öffentlichen Beihilfen, Einkommen aus Vermietung und Verpachtung, Wohngeld, Kindergeld und sonstige Einkünfte wie Kapitalerträge, abzüglich Steuern und Sozialversicherungsbeiträgen. Die Befragten konnten sich einer von 9 Gehaltsgruppen zuordnen. Zur besseren Vergleichbarkeit der Einkommensgruppen wurden die Kategorien des Fragebogens für die vorliegende Untersuchung mit gleicher Breite zusammengefasst. Eine Berechnung des Äquivalenzeinkommens als individueller Einkommenswert war anhand der zur Verfügung stehenden Daten in methodisch belastbarer Weise nicht möglich.

#### Bildung.

Der Bildungsstatus wurde über den formal höchsten Bildungsabschluss definiert. Auch hier konnten die Befragten per Selbstauskunft aus 6 Antwortoptionen auswählen: Hauptschulabschuss, Realschulabschluss, Fachhochschulreife, Abitur, Hochschulabschluss und Promotion. Zur besseren Übersicht und aufgrund kleiner Teilstichproben wurden für die Auswertung die Kategorien Fachhochschulreife und Abitur sowie Hochschulabschluss und Promotion zusammengefasst.

### Merkmale des Pflegearrangements

Im Fokus der hier vorgestellten Sekundäranalyse steht die *Nutzung von Unterstützungsleistungen* für pflegebedürftige Menschen. Der Fragebogen erfasste dazu Angaben zur Nutzung folgender Leistungen:

- Pflegegeld,
- ambulante Pflege durch einen Pflegedienst,
- Tages‑/Nachtpflege,
- Verhinderungs‑/Ersatzpflege,
- Kurzzeitpflege,
- Entlastungsbetrag,
- Haushaltshilfe,
- Betreuungsdienste,
- „24-Stunden-Pflege“.

Damit werden die relevantesten Leistungsangebote zur Unterstützung häuslicher Pflege untersucht. Die Nutzung wurde als Mehrfachantwort abgefragt („Welche der folgenden Leistungen nehmen Sie/nimmt die pflegebedürftige Person in Anspruch?“). Zusätzlich wurde auch der *Umfang in Anspruch genommener Unterstützungsleistungen* in Zeitwerten abgefragt, etwa wie lange sich der Pflegedienst täglich im Haushalt der pflegebedürftigen Person aufhält. Die Mehrheit der aufgeführten Unterstützungsleistungen werden von der Pflegeversicherung mitfinanziert (ambulante Pflege durch einen Pflegedienst, Tages‑/Nachtpflege, Verhinderungs‑/Ersatzpflege, Kurzzeitpflege) oder sind direkte Geldleistungen der Pflegeversicherung (Pflegegeld, Entlastungsbetrag). Haushaltshilfen und Betreuungsdienste können mithilfe des Entlastungsbetrags (teil‑)finanziert werden.

Weiterhin wurde die Nutzung von *Beratungsangeboten* sowie *wohnraumanpassender Maßnahmen* erfasst („Haben Sie sich schon einmal zu Pflegefragen beraten lassen?“ bzw. „Haben Sie wegen der Pflege im Haus/in der Wohnung Veränderungen oder Umbauarbeiten vorgenommen?“). Beratungsangebote sind vollständig von der Pflegeversicherung getragen, allerdings liegen Hinweise vor, dass deren Nutzung Bildungseffekten unterliegt [30]. Wohnraumanpassende Maßnahmen erfordern in aller Regel eine größere finanzielle Eigenleistung der Betroffenen, sind aber nicht selten entscheidend für die Sicherung der häuslichen Pflege [31]. Daher werden beide Angebote in die Analyse einbezogen.

Die Befragten wurden zudem gebeten *einzuschätzen*, wie sie ihre Pflegesituation erleben („Wie schätzen Sie Ihre Pflegesituation insgesamt ein?“). Die Antwortoptionen reichten von „sehr gut zu bewältigen“ bis „eigentlich gar nicht mehr zu bewältigen“. Wenngleich dies nicht in direktem Bezug zur Fragestellung der Gestaltung häuslicher Pflegearrangements steht, ermöglicht eine Auswertung Rückschlüsse auf die subjektive Bewertung der Pflegesituation durch die pflegebedürftigen und pflegenden Personen.

### Statistische Auswertung

Die statistischen Analysen (IBM® SPSS® Statistics 26, IBM, Armonk, NY, USA) umfassten deskriptive Auswertungen sowie die Untersuchung von Zusammenhängen zwischen den sozioökonomischen Merkmalen einerseits und den Merkmalen des Pflegearrangements andererseits (vgl. Onlinematerial 1: Zusammenfassung erstellter Statistik). Die Zusammenhangsanalyse erfolgte anhand von Chi-Quadrat-Tests und Spearman-Rangkorrelationsanalysen. Das Signifikanzniveau wurde bei α = 0,05 festgelegt. Zur besseren Interpretierbarkeit der Ergebnisse wurden *Cramers V *sowie der Spearman-Korrelationskoeffizient *r*_*s*_ als Parameter zur Darstellung der Effektstärke herangezogen. Die Interpretation der Effektstärke erfolgte analog zu den Empfehlungen von Cohen [32], so dass Werte unter 0,1 sowohl für *Cramers V* als auch *r*_*s*_ als vernachlässigbar eingeordnet werden.

Die aufgeführten Analysen wurden zusätzlich differenziert nach Pflegegrad, Beziehungsstatus zwischen pflegebedürftiger und pflegender Person und dem Status als Hauptpflegeperson, da anzunehmen ist, dass diese Merkmale jeweils unabhängig des sozialen Status Einfluss auf die Nutzung von Unterstützungsleistungen nehmen.

## Ergebnisse

### Stichprobenbeschreibung

Eine Übersicht der Stichprobe findet sich in Tab. 1 und Tab. 2 (für eine detaillierte Beschreibung vgl. auch Onlinematerial 2: Stichprobenbeschreibung). In der Gruppe der befragten pflegebedürftigen Personen (*n* = 3871) entspricht die Konzentration der Stichprobe auf die Pflegegrade 2 und 3 den Daten der Pflegestatistik^1^ [4]. Pflegebedürftige mit dem Pflegegrad 1 sind leicht über- und Anspruchsberechtigte mit den Pflegegraden 4 und 5 etwas unterrepräsentiert. Wie aus Tab. 1 ersichtlich konzentriert sich die Altersverteilung in der analysierten Stichprobe der pflegebedürftigen Personen auf die Altersklassen 55 bis 60 Jahre sowie 61 bis 65 Jahre, die zusammen fast die Hälfte der Befragten bilden (47,6 %). Hingegen sind Pflegebedürftige ab einem Alter von 81 Jahren mit nur 13,4 % vertreten. Damit sind jüngere Jahrgänge im Vergleich zur Pflegestatistik stark überrepräsentiert: Hier liegt die Konzentration der Pflegebedürftigkeit mit einem Anteil von 64 % auf Menschen ab 75 Jahre. Jeweils die Hälfte der befragten pflegebedürftigen Personen ist männlich bzw. weiblich. Die Pflegestatistik weist dagegen mit etwas mehr als 60 % einen höheren Anteil weiblicher Pflegebedürftiger aus.

**Table 1:** 

Pflegebedürftige Personen nach SGB XI ab 55 Jahren, *n* = 3871		Anteil in Prozent
*Personenbezogene Merkmale der pflegebedürftigen Person*		
Alter (Pflichtmerkmal, *n* = 3871)	91 Jahre und älter	1,7
	86 bis 90 Jahre	2,7
	81 bis 85 Jahre	9
	76 bis 80 Jahre	9,9
	71 bis 75 Jahre	13,6
	66 bis 70 Jahre	15,6
	61 bis 65 Jahre	22,1
	55 bis 60 Jahre	25,5
Pflegegrad (Pflichtmerkmal, *n* = 3871)	Pflegegrad 1	15,4
	Pflegegrad 2	44,9
	Pflegegrad 3	29,0
	Pflegegrad 4	8,7
	Pflegegrad 5	2,1
Geschlecht (*n* = 3216)	Männlich	49,8
	Weiblich	50,0
*Sozioökonomische Merkmale der pflegebedürftigen Person*		
Haushaltsnettoeinkommen (*n* = 3433)	Weniger als 1000 €	11,7
	1000 € bis 1999 €	40,1
	2000 € bis 2999 €	28,7
	3000 € bis 3999 €	11,6
	Mehr als 4000 €	7,9
Höchster Bildungsabschluss (*n* = 3150)	Hauptschulabschluss	28,8
	Realschulabschluss	29,1
	Fachhochschulreife/Abitur	24,7
	Hochschulabschluss/Promotion	17,4

*SGB XI* Elftes Buch Sozialgesetzbuch

**Table 2:** 

Pflegepersonen von pflegebedürftigen Personen nach SGB XI ab 55 Jahren, *n* = 17.990		Anteil in Prozent
*Personenbezogene Merkmale der Pflegeperson und der pflegebedürftigen Person*		
Alter der befragten Person (Pflegeperson; *n* = 13.867)	76 bis 95 Jahre	6,1
	66 bis 75 Jahre	19,3
	56 bis 65 Jahre	52,7
	46 bis 55 Jahre	18,1
	16 bis 45 Jahre	3,7
Geschlecht der befragten Person (*n* = 13.970)	Männlich	29,8
	Weiblich	70,2
Beziehung zur pflegebedürftigen Person (*n* = 17.939)	Mein Ehepartner/mein Lebensgefährte	23,5
	Mein Vater/meine Mutter	57,2
	Anderes Verwandtschaftsverhältnis	15,1
	Ein Freund/Nachbar	2,3
	Sonstige	1,8
Befragte Person ist Hauptpflegeperson (17.490)	Ja	75,0
	Nein	25,0
Pflegegrad der pflegebedürftigen Person (Pflichtmerkmal; *n* = 17.990)	Pflegegrad 1	5,3
	Pflegegrad 2	27,0
	Pflegegrad 3	35,5
	Pflegegrad 4	21,0
	Pflegegrad 5	11,2
*Sozioökonomische Merkmale der Pflegeperson*		
Haushaltsnettoeinkommen (*n* = 11.991)	Weniger als 1000 €	7,9
	1000 € bis 1999 €	30,1
	2000 € bis 2999 €	33,7
	3000 € bis 3999 €	16,2
	Mehr als 4000 €	12,1
Höchster Bildungsabschluss (*n* = 13.477)	Hauptschulabschluss	22,7
	Realschulabschluss	35,0
	Fachhochschulreife/Abitur	25,6
	Hochschulabschluss/Promotion	16,7

*SGB XI* Elftes Buch Sozialgesetzbuch

In der Stichprobe der Pflegepersonen (*n* = 17.990) betreuen über 32 % der Befragten eine pflegebedürftige Person mit Pflegegrad 4 oder 5. In dieser Stichprobe sind damit Pflegearrangements in Schwer- und Schwerstpflegebedürftigkeit überrepräsentiert. Von den befragten Pflegepersonen sind 70,2 % weiblich, was bisherigen Befunden der überwiegend weiblich geprägten Pflegeübernahme entspricht (vgl. etwa [33]). Fast 80 % der befragten Pflegepersonen sind 56 Jahre oder älter, der Altersmittelwert liegt bei 62 Jahren. Damit sind die Befragten durchschnittlich etwas älter als andere Studien dies ausweisen [34].

Wie Tab. 1 zu entnehmen ist, geben gut 2 Drittel der befragten Pflegebedürftigen ein Haushaltsnettoeinkommen zwischen 1000 € und 3000 € an, wobei die Mehrheit (40,1 %) auf die Einkommenskategorie 1000 € bis 1999 € entfällt. Pflegepersonen ordnen ihr Haushaltseinkommen etwas höher ein, wobei auch hier der größte Anteil in den Einkommenskategorien 1000 € bis 1999 € sowie 2000 € bis 2999 € zu finden ist (Tab. 2). Damit liegt in der vorliegenden Stichprobe das angegebene Einkommen mehrheitlich unter dem bundesweiten Durchschnitt von ca. 3600 € [35, 36].

Die befragten pflegebedürftigen Personen verfügen mit knapp 60 % mehrheitlich über einen Hauptschul- oder Realschulabschluss, gut 17 % haben mindestens einen Hochschulabschluss. Der Bildungsstatus dieser Teilstichprobe ist damit vergleichbar mit dem bundesdeutschen Durchschnitt in der Altersgruppe 60 bis 65 Jahre [37]. Die befragten Pflegepersonen verfügen ebenfalls mehrheitlich über einen Haupt- oder Realschulabschluss, den etwas mehr als 25 % haben. Für die Gruppe der Pflegepersonen muss umso mehr noch die Altersspanne der Befragten berücksichtigt werden, da über die Generationen hinweg deutliche Bildungsunterschiede zu verzeichnen sind. Unter Betrachtung der Personen zwischen 60–65 Jahren sind die statistischen Zahlen der Stichprobe mit dem Bevölkerungsdurchschnitt vergleichbar.

Im Folgenden werden jene Ergebnisse der Auswertung berichtet, die statistisch signifikant sind und eine relevante Effektgröße aufweisen. Eine detaillierte Ergebnisübersicht zu den vorgenommenen Analysen findet sich im Onlinematerial 3: Ergebnisse aller durchgeführten statistischen Analysen.

### Inanspruchnahme von Unterstützungsangeboten

In der Gruppe der befragten pflegebedürftigen Menschen zeigt sich ein signifikanter Zusammenhang zwischen dem Einkommen und der Inanspruchnahme von Pflegegeld, der aber knapp das Niveau relevanter Effektstärke unterschreitet (χ^2^(4) = 25,537, *p* < 0,001, *n* = 2738, *Cramers V* = 0,097). Die Richtung des Zusammenhangs besteht entgegen bisheriger empirischer Erkenntnisse: Pflegegeld wird mit steigendem Einkommen etwas häufiger genutzt. Weiterhin zeigt sich hinsichtlich des Zusammenhangs zwischen dem Einkommen pflegebedürftiger Personen und der Nutzung einer sogenannten „24-Stunden-Pflege“ ein signifikanter, aber hinsichtlich der Effektstärke vernachlässigbarer Zusammenhang (χ^2^(4) = 15,669, *p* = 0,003, *n* = 3241, *Cramers V* = 0,070).

Ein statistisch relevanter Zusammenhang zwischen dem Einkommen von Pflegepersonen und der Nutzung der benannten Unterstützungsleistungen kann nicht festgestellt werden. Ebenso kann in beiden Teilstichproben ein Zusammenhang mit dem Bildungsstatus nicht nachgewiesen werden.

#### Umfang in Anspruch genommener Unterstützungsleistungen.

**Figure 1:**
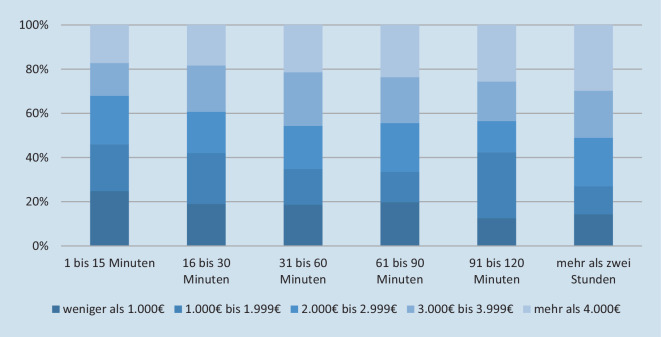

Hinsichtlich des Umfangs der genutzten Unterstützungsleistungen besteht ein signifikanter Zusammenhang zwischen dem Einkommen der pflegebedürftigen Person und dem Umfang der Nutzung eines Pflegedienstes (*r*_*s*_ = 0,081, *p* = 0,021, *n* = 630) sowie einer Haushaltshilfe (*r*_*s*_ = 0,072, *p* = 0,007, *n* = 1136). Beide Ergebnisse müssen aber bei nicht vorhandener Effektstärke als nicht aussagekräftig verworfen werden. Beim direkten Vergleich der obersten und untersten Einkommensgruppe wird allerdings deutlich, dass zumindest zwischen diesen beiden Einkommensgruppen deutliche Unterschiede im Umfang genutzter Unterstützungsleistungen bestehen (vgl. zur Nutzung eines Pflegedienstes Abb. 1).

Bei differenzierter Betrachtung der Teilstichprobe der Pflegepersonen wird deutlich, dass insbesondere innerhalb einer (Ehe‑)Partnerschaft mit zunehmendem Einkommen eine Haushaltshilfe in höherem Umfang finanziert wird (*r*_*s*_ = 0,177, *p* < 0,001, *n* = 781). Zusammenhänge zeigen sich auch, wenn nur befragte Hauptpflegepersonen betrachtet werden: Hier stehen sowohl ein höheres Einkommen (*r*_*s*_ = 0,146, *p* < 0,001, *n* = 2243) als auch ein höherer Bildungsstatus (*r*_*s*_ = 0,121, *p* < 0,001, *n* = 2475) signifikant mit einem höheren wöchentlichen Umfang von Unterstützung im Haushalt in Zusammenhang. Ein signifikantes Ergebnis mit vernachlässigbarem Effekt zeigt sich bei der Untersuchung des Zusammenhangs zwischen Einkommen und Bildung der Hauptpflegeperson mit dem Umfang genutzter formeller Unterstützung durch einen Pflegedienst (Einkommen: *r*_*s*_ = 0,057, *p* = 0,001, *n* = 3311; Bildung: *r*_*s*_ = 0,051, *p* = 0,001, *n* = 3705).

### Inanspruchnahme von Beratungsangeboten

Ein Zusammenhang zwischen dem Einkommen bzw. dem Bildungsniveau pflegebedürftiger Personen mit der Inanspruchnahme von Beratungsangeboten kann in der Stichprobe nicht festgestellt werden. Das Bildungsniveau von Pflegepersonen steht dagegen mit nennenswertem Effekt im Zusammenhang mit der Nutzung von Beratung zu Pflegefragen. Die Inanspruchnahme von Beratungsangeboten steigt insbesondere dann mit dem Bildungsniveau, wenn ein enger Beziehungsstatus mit der pflegebedürftigen Person besteht (Partner*in: χ^2^(3) = 48,009, *p* < 0,001, *n* = 3305, *Cramers V* = 0,121; Mutter/Vater: χ^2^(3) = 65,465, *p* < 0,001, *n* = 7301, *Cramers V* = 0,097).

### Inanspruchnahme von Maßnahmen der Wohnraumanpassung

**Figure 2:**
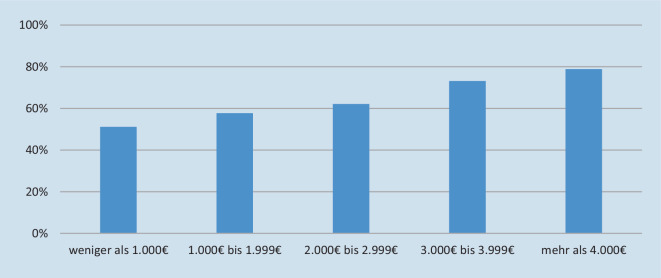

Maßnahmen zur Wohnraumanpassung stehen in signifikantem Zusammenhang mit dem Einkommen der pflegebedürftigen Person (χ^2^(4) = 87,892, *p* < 0,001, *n* = 3121, *Cramers V* = 0,168; Abb. 2), nicht aber mit jenem von Pflegepersonen. Auch das Einkommen pflegender Partner*innen steht in der Stichprobe in keinem Zusammenhang mit der Durchführung wohnraumanpassender Maßnahmen, wobei das Effektmaß *V* nur knapp noch im Bereich des Vernachlässigbaren liegt (χ^2^(4) = 26,831, *p* < 0,001, *n* = 3060, *Cramers V* = 0,094).

### Einschätzung der Pflegesituation

**Figure 3:**
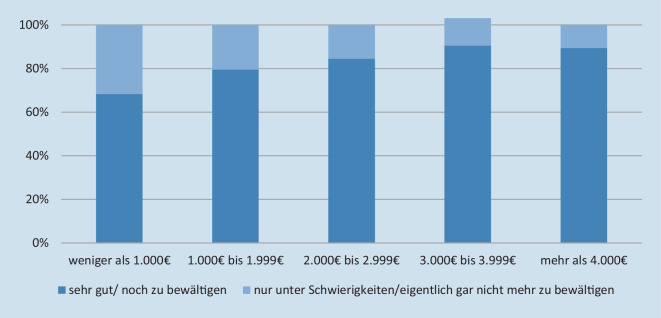

Die Einschätzung der Pflegesituation steht in der Gruppe der pflegebedürftigen Personen im Zusammenhang mit der Einkommenssituation: Je niedriger das Einkommen, desto häufiger wird die Pflegesituation als nur „unter Schwierigkeiten“ oder als „eigentlich gar nicht mehr zu bewältigen“ eingeschätzt (*r*_*s*_ = −0,142, *p* < 0,001, *n* = 3045; Abb. 3). Dieser Effekt zeigt sich insbesondere bei Pflegegrad 1 (*r*_*s*_ = −0,198, *p* < 0,001, *n* = 474). Bei den Pflegegraden 4 (*n* = 249) und 5 (*n* = 64) lässt sich in der kleinen Stichprobe kein signifikanter Zusammenhang feststellen.

In der Gruppe der Pflegepersonen ist der Zusammenhang zwischen einer negativen Bewertung der Pflegesituation und einem geringen Einkommen signifikant, aber mit nicht vorhandener Effektstärke (*r*_*s*_ = −0,031, *p* = 0,001, *n* = 11.527). Bemerkenswert erscheint die Tendenz, dass mit sinkendem Bildungsniveau der Hauptpflegeperson die Pflegesituation positiver eingeschätzt wird, sich also ein zum Einkommenseffekt gegenläufiger Zusammenhang andeutet (*r*_*s*_ = 0,095, *p* < 0,001, *n* = 9863).

## Diskussion

Die *Nutzung von Unterstützungsleistungen *scheint anhand der Ergebnisse der vorliegenden Studie nicht in nennenswertem Maß mit dem Einkommen von pflegebedürftigen Personen zu korrespondieren, auch mit dem Bildungsstatus zeigt sich kein Zusammenhang. Dies entspricht bisherigen Befunden, denen zufolge sich die Entscheidung zum Zukauf von Unterstützungsleistungen in erster Linie am Verlauf des Pflegebedarfs orientiert und hohes Einkommen nur leicht mit höherer Inanspruchnahme von professionellen Pflegeleistungen assoziiert ist [24, 25]. Weiter verfolgt werden muss allerdings die Erkenntnis, dass die Nutzung von Pflegegeld mit steigendem Einkommen leicht zunimmt. Sollte dieser Befund aus der analysierten Datenlage über vertiefende Studien bestätigt werden, wäre dies ein Hinweis darauf, dass Pflegegeld insbesondere jene nicht unterstützt, die am meisten davon profitieren würden.

Hinsichtlich des *Umfangs genutzter Unterstützungsleistungen* zeigt sich ein vorsichtig zu interpretierender Zusammenhang zwischen dem Einkommen und der Nutzung eines Pflegedienstes. Dies erscheint insofern plausibel, als bei zurückhaltender Nutzung eines Pflegedienstes es entweder keiner weiteren Zuzahlung über den von der Pflegeversicherung gezahlten Leistungsbetrag hinaus bedarf oder sogar noch ein verbleibender Restbetrag (Kombinationsleistung) ausgezahlt werden kann.

Sichtbar wird auch der mit steigendem Einkommen zunehmende Umfang der Unterstützung durch eine Haushaltshilfe sowie die Umsetzung wohnraumanpassender Maßnahmen. Es liegt die Vermutung nahe, dass sich einkommensschwächere Pflegehaushalte auf die Finanzierung von Pflegeleistungen konzentrieren und eher als einkommensstarke Haushalte auf darüber hinaus mögliche Leistungen zur Erleichterung der Pflegesituation verzichten wollen oder müssen. Da sowohl Unterstützung im Haushalt als auch die pflegegerechte Gestaltung des Wohnraums die Pflege erleichtern und die Qualität der häuslichen Versorgung erhöhen können [38], geht dieser Verzicht möglicherweise mit negativen Konsequenzen für die Lebbarkeit häuslicher Pflegearrangements in einkommensschwachen Pflegehaushalten einher.

Bei der *Nutzung von Beratungsangeboten* zeigt sich ein Zusammenhang mit dem Bildungsstatus der Pflegeperson, was bisherigen Befunden entspricht [26, 30]. Eine Konzentration der Inanspruchnahme von Beratungsangeboten auf höhere Bildungsgruppen kann damit zum derzeitigen Zeitpunkt als gesichert bezeichnet werden. Dieser Befund ist damit nicht neu, sollte allerdings vor dem Hintergrund, dass Beratung entscheidend zur Qualität und Stabilität häuslicher Pflegearrangements beitragen kann [39], Implikationen für eine zielgruppengerechtere Ansprache der beratenden Institutionen nach sich ziehen. Gleichzeitig zeigt sich in der Stichprobe eine insgesamt starke Nutzung von Beratung, was auf eine positive Entwicklung der Beratungsstrukturen hindeutet.

Die *Einschätzung der Pflegesituation* fällt bei 80 % der befragten pflegebedürftigen Personen insgesamt positiv aus. Auf der anderen Seite empfinden immerhin 20 % nennenswerte Schwierigkeiten in der Bewältigung der Pflege, und dies ist umso häufiger der Fall, je niedriger das Einkommen der Betroffenen ist. Eine ungleiche Verteilung von Unterstützungsleistungen scheint diesen Befund wie dargestellt nicht begründen zu können. Unter Bezugnahme auf Arbeiten von Heusinger [40, 41] wäre ein möglicher Ansatzpunkt die Auseinandersetzung mit ungleichen Möglichkeiten sozialer Teilhabe, der für pflegebedürftige Personen aus niedrigen Statusgruppen als kritischer Punkt herausgearbeitet wurde, in der vorliegenden Studie aber nicht abgebildet werden kann. Ein weiterer möglicher Aspekt kann darin gesucht werden, dass nach Finanzierung der Pflege für einkommensschwache Haushalte im Verhältnis weniger Geld zur Lebensgestaltung übrigbleibt (vgl. auch [26]), was die pessimistischere Sicht auf die Pflegesituation plausibel erscheinen lässt. Hier gilt es, das Forschungsfeld weiter auf die Frage zu fokussieren, ob die kritischere Bewertung der Pflegesituation Ausdruck einer allgemein pessimistischeren Lebensperspektive ist oder möglicherweise andere Parameter als die hier erfassten zur Beschreibung von Ungleichheit in der häuslichen Pflege herangezogen werden müssen.

### Limitationen

Einschränkungen hinsichtlich der Belastbarkeit der Ergebnisse ergeben sich aus der Stichprobe, die unter statistischen Gesichtspunkten nicht repräsentativ ist, jedoch in relevanten Merkmalen dem Durchschnitt der bekannten Grundgesamtheit entspricht. Die vorgestellten Erkenntnisse sind vor diesem Hintergrund zunächst als Hinweise zu interpretieren, die durch weitere Studien zu prüfen sind. Dabei wäre durch multivariate Analysen insbesondere auch zu untersuchen, inwiefern weitere pflegebeeinflussende Aspekte wie chronische Erkrankungen und Multimorbidität, die überproportional in sozioökonomisch schwächeren Bevölkerungsgruppen anzutreffen sind [3, 10], Einfluss auf die Ergebnisse nehmen.

Limitationen ergeben sich auch hinsichtlich der Erfassung der sozioökonomischen Merkmale Einkommen und Bildung. Da die Abfrage des Haushaltseinkommens kategorial erfolgte und die Altersstruktur weiterer Haushaltsmitglieder nicht abgefragt wurde, konnte das Äquivalenzeinkommen (Einkommen pro Kopf) methodisch nicht belastbar berechnet werden [42]. Es bleibt zu prüfen, ob diese Limitationen ggf. für den geringen Effekt des Einkommens auf die Inanspruchnahme von 24-Stunden-Pflege verantwortlich sind, da diese Erkenntnis der bisherigen Studienlage widerspricht [24, 26].

Die berufliche Bildung wurde abseits akademischer Abschlüsse nicht bei der Angabe des höchsten Bildungsabschlusses berücksichtigt (vgl. dazu [43]). Der tertiäre Bildungsbereich (etwa Fachschulen und Berufsakademien) ist demnach nicht vollständig erfasst. Gleichzeitig bot der Fragebogen nicht die Möglichkeit anzugeben, dass kein Schulabschluss vorliegt, was immerhin auf 4 % der Bevölkerung zutrifft [44]. Der in dieser Studie nicht nachweisbare Zusammenhang von Bildung und der Gestaltung häuslicher Pflegearrangements muss daher mit Zurückhaltung interpretiert werden.

Die Limitationen der Studie zeigen insbesondere auf, mit welchen Herausforderungen die Untersuchung sozioökonomischer Einflüsse auf die Gestaltung der häuslichen Pflege behaftet ist. Dies beginnt grundlegend mit der Erfassung der Einkommens- und Bildungssituation, die methodisch als durchaus komplex zu bewerten ist, und geht weiter mit der noch zu beantwortenden Frage, welche Parameter Ungleichheiten im häuslichen Pflegearrangement definieren.

## Fazit

Die Ausgestaltung der Pflegeversicherung als Teilleistungsversicherung sowie die Komplexität der Versorgungslandschaft begründen die Annahme, dass die Nutzung von Versorgungsangeboten von finanziellen und Bildungsressourcen beeinflusst wird. Dies lässt sich mit der vorliegenden Studie jedoch nicht eindeutig empirisch belegen. Der Widerspruch zu vorliegenden qualitativen Arbeiten, die deutlich negative Einflüsse einer benachteiligten Einkommenssituation auf die Pflege herausarbeiten, bleibt damit weiterhin unaufgelöst. Dennoch weisen die Ergebnisse in der Gesamtschau auf Tendenzen hin, die zu diskutieren sind.

Zumindest die Leistungen, die über die Pflegeversicherung (mit‑)finanziert werden, weisen in der hier vorliegenden Studie keine erheblichen Ungleichheiten in der Nutzung auf, von Maßnahmen der Wohnraumanpassung und dem Umfang von Unterstützung im Haushalt einmal abgesehen. Unterschiede zwischen den Einkommensgruppen zeigen sich aber in der Bewertung der Pflegesituation, die in niedrigeren Einkommensgruppen eher als nicht oder nur schlecht bewältigbar erlebt wird. Angesichts der zunehmenden Anzahl pflegebedürftiger Menschen wird kontinuierlich die Frage zu stellen sein, welchen Einfluss sozioökonomische Faktoren einerseits auf Pflegeentscheidungen und -präferenzen innerhalb von Familien und sozialen Netzwerken haben und ob das Unterstützungssystem der Pflegeversicherung mögliche Ungleichheiten verstärkt.

## Supplementary Information







## Footnotes

1 Die Pflegestatistik differenziert nach Anspruchsberechtigten, die „zu Hause versorgt“ werden, und solchen, die in vollstationären Einrichtungen betreut werden. Für die Einordnung der analysierten Stichprobe wurden aus der Pflegestatistik die Daten für zu Hause versorgte Pflegebedürftige herangezogen.
