# Supplementary material for: Homecare arrangements: examining the use of support services in relation to socioeconomic status
Source: Bundesgesundheitsblatt Gesundheitsforschung Gesundheitsschutz. 2023 Mar 20;66(5):540–9. [Article in German] doi: 10.1007/s00103-023-03684-6 (PMC10163119; doi:10.1007/s00103-023-03684-6)
Supplement: Supplementary file 1 [file 103_2023_3684_MOESM1_ESM.pdf]

Onlinematerial 1

Englert et al. (2023): Häusliche Pflegearrangements: Untersuchung der Inanspruchnahme von Unterstützungsleistungen im Zusammenhang mit dem sozioökonomischen Status

-Zusammenfassung erstellter Statistik (Summary of Statistics)-

Stichprobe: Pflegebedürftige Personen nach SGB XI ab 55 Jahre				
	**Variablenname**	**Beschreibung/Frage**	**Ausprägungen/Codierung**	**Durchgeführter Test**
**Unabhängige Variablen** (Sozio-ökonomische Merkmale)	Haushaltseinkommen (HHE)	1.21 Wie hoch ist ungefähr das monatliche Netto-Einkommen?	1=weniger als 1.000€ / 2=1.000€ - 1.999€ / 3=2.000€ - 2.999€/ 4= 3.000€ - 3.999€/ 5=4.000€ und mehr	
	Bildungsabschluss	1.20 Welchen höchsten Bildungsabschluss haben Sie?	1=Hauptschulabschluss/ 2=Realschulabschluss/ 3=Fachhochschulreife/Abitur/ 4= Hochschulabschluss Promotion	
**Abhängige Variablen** (Merkmale des Pflege-arrangements)	**Nutzung von Unterstützungsleistungen**			
	Pflegegeld	3.1 Bei den nächsten Fragen geht es um Unterstützungsleistungen für pflegebedürftige Menschen. Welche der folgenden Leistungen nehmen Sie in Anspruch? *Mehrfachantwort*	0=Nicht gewählt; 1=Ja(Gewählt)	Chi2 nach Pearson; Cramer V (bei signifikantem Ergebnis >0,05)
	Ambulanter Pflegedienst			
	Tages-/Nachtpflege			
	Verhinderungspflege			
	Kurzzeitpflege			
	Entlastungsbetrag			
	Haushaltshilfe			
	Betreuungsdienst			
	24-Stunden-Pflege			
	**Umfang genutzter Unterstützungsleistungen**			
	Pflegedienst Umfang	3.5 Wie lange ist der Pflegedienst täglich bei Ihnen?	1=1 bis 15 Minuten; 2=16 bis 30 Minuten; 3=31 bis 60 Minuten; 4=61 bis 90 Minuten; 5=91 bis 120 Minuten; 6=mehr als zwei Stunden	Rangkorrelation nach Spearman (einseitig/gerichtet)
	Tagespflege/Nachtpflege Umfang	3.6 Für wie viele Stunden in der Woche nehmen Sie Tages- oder Nachtpflege in Anspruch?	1=1 bis 8 Stunden; 2=9 bis 16 Stunden; 3=17 bis 24; 4=25 bis 32 Stunden; 5=33 bis 40 Stunden; 6=mehr als 40 Stunden	
	Verhinderungspflege Umfang Stunden/Woche	3.7.1 Wie viele Stunden wöchentlich haben Sie im Durchschnitt in den letzten 12 Monaten Verhinderungs-, Ersatzpflege genutzt?	1=1 bis 24 Stunden; 2=25 bis 48 Stunden; 3=49 bis 72 Stunden; 4=73 bis 96 Stunden; 5=97 bis 120 Stunden; 6=121 bis 144 Stunden; 7=144 bis 168 Stunden	
	Verhinderungspflege Umfang Wochen/Jahr	3.7.2 Wenn Sie in den letzten 12 Monaten Verhinderungs-, Ersatzpflege für einen längeren Zeitraum am Stück in Anspruch genommen haben, wie viele Wochen waren das?	1= bis 1 Woche; 2= mehr als 1 Woche bis 2 Wochen; 3= mehr als 2 Wochen bis 3 Wochen; 4= mehr als 3 Wochen bis 6 Wochen; 5= mehr als 6 Wochen bis 12 Wochen; 6=mehr als 12 Wochen	
	Kurzzeitpflege Umfang	3.8 Wie viele Wochen haben sie in den letzten 12 Monaten Kurzzeitpflege in Anspruch genommen?	1=bis zu einer Woche; 2=bis zu zwei Wochen; 3=bis zu drei Wochen; 4=bis zu vier Wochen; 5=bis zu fünf Wochen; 6=bis zu sechs Wochen; 7=mehr als 6 Wochen	
	Haushaltshilfe Umfang	3.10 Wie viele Stunden in der Woche nutzen Sie eine Haushaltshilfe (Kochen, Putzen, Einkaufe)?	1=bis zu einer Stunde; 2=mehr als eine Stunde; 3=mehr als zwei Stunden; 4=mehr als drei Stunden; 5=mehr als vier Stunden	

	Betreuungsdienste Umfang	3.11 Wie viele Stunden in der Woche nutzen Sie einen Betreuungsdienst?	1=bis zu einer Stunde; 2=mehr als eine Stunde; 3=mehr als zwei Stunden; 4=mehr als drei Stunden; 5=mehr als vier Stunden	Rangkorrelation nach Spearman (einseitig/gerichtet)
	**Wohnraumanpassende Maßnahmen**			
	Wohnraumanpassung	4.2 Haben Sie wegen der Pflege im Haus/ in der Wohnung Veränderungen oder Umbauarbeiten vorgenommen?	1=Ja; 2=Nein	Chi2 nach Pearson; Cramer V (bei signifikantem Ergebnis >0,05)
	**Nutzung von Beratungsangeboten**			
	Beratung genutzt	5.0 Haben Sie sich schon einmal zu Pflegefragen beraten lassen?	10=Nein; 9=Ja	Chi2 nach Pearson; Cramer V (bei signifikantem Ergebnis >0,05)
	**Einschätzung der Pflegesituation**			
	Einschätzung Pflegesituation	7.1 Wie schätzen Sie Ihre Pflegesituation insgesamt ein? Die häusliche Pflege ist…	1=sehr gut zu bewältigen; 2=noch zu bewältigen; 3=nur unter Schwierigkeiten zu bewältigen; 4=eigentlich gar nicht mehr zu bewältigen	Rangkorrelation nach Spearman (einseitig/gerichtet)

Stichprobe: Pflegepersonen von pflegebedürftigen Personen nach SGB XI ab 55 Jahre				
	**Variablenname**	**Beschreibung/Frage**	**Ausprägungen/Codierung**	**Durchgeführter Test**
**Unabhängige Variablen** (Sozio-ökonomische Merkmale)	Haushaltseinkommen	1.21 Wie hoch ist ungefähr das monatliche Netto-Einkommen?	1=weniger als 1.000€ / 2=1.000€ - 1.999€ / 3=2.000€ - 2.999€/ 4= 3.000€ - 3.999€/ 5=4.000€ und mehr	
	Bildungsabschluss	1.20 Welchen höchsten Bildungsabschluss haben Sie?	1=Hauptschulabschluss/ 2=Realschulabschluss/ 3=Fachhochschulreife/Abitur/ 4= Hochschulabschluss Promotion	
**Abhängige Variablen** (Merkmale des Pflege-arrangements)	**Nutzung von Unterstützungsleistungen**			
	Pflegegeld<br>Ambulanter Pflegedienst<br>Tages-/Nachtpflege<br>Verhinderungspflege<br>Kurzzeitpflege<br>Entlastungsbetrag<br>Haushaltshilfe<br>Betreuungsdienst<br>24-Stunden-Pflege	4.1 Bei den nächsten Fragen geht es um Unterstützungsleistungen für pflegebedürftige Menschen. Welche der folgenden Leistungen nimmt die pflegebedürftige Person in Anspruch? *Mehrfachnennung möglich*	0=Nicht gewählt; 1=Ja (Gewählt)	Chi2 nach Pearson; Cramer V (bei signifikantem Ergebnis >0,05)
	**Umfang genutzter Unterstützungsleistungen**			
	Pflegedienst Umfang	4.5 Wie lange ist der Pflegedienst täglich im Haushalt der pflegebedürftigen Person? Wenn Sie es nicht genau wissen, schätzen Sie bitte.	1=1 bis 15 Minuten; 2=16 bis 30 Minuten; 3=31 bis 60 Minuten; 4=61 bis 90 Minuten; 5=91 bis 120 Minuten; 6=mehr als zwei Stunden	Rangkorrelation nach Spearman
	Tages-/Nachtpflege Umfang	4.6 Für wie viele Stunden in der Woche nimmt die pflegebedürftige Person Tages- oder Nachtpflege in Anspruch?	1=1 bis 8 Stunden; 2=9 bis 16 Stunden; 3=17 bis 24; 4=25 bis 32 Stunden; 5=33 bis 40 Stunden; 6=mehr als 40 Stunden	
	Verhinderungspflege Umfang Stunden/Woche	4.7.1 Wie viele Stunden wöchentlich haben Sie im Durchschnitt in den letzten 12 Monaten Verhinderungs-, Ersatzpflege genutzt?	1=1 bis 24 Stunden; 2=25 bis 48 Stunden; 3=49 bis 72 Stunden; 4=73 bis 96 Stunden; 5=97 bis 120 Stunden; 6=121 bis 144 Stunden; 7=144 bis 168 Stunden	
	Verhinderungspflege Umfang Wochen/Jahr	4.7.2 Wenn Sie in den letzten 12 Monaten Verhinderungs-, Ersatzpflege für einen längeren Zeitraum am Stück in Anspruch genommen haben, wie viele Wochen waren das?	1= bis 1 Woche; 2= mehr als 1 Woche bis 2 Wochen; 3= mehr als 2 Wochen bis 3 Wochen; 4= mehr als 3 Wochen bis 6 Wochen; 5= mehr als 6 Wochen bis 12 Wochen; 6=mehr als 12 Wochen	
	Kurzzeitpflege Umfang	4.8 Wie viele Wochen haben sie in den letzten 12 Monaten Kurzzeitpflege in Anspruch genommen?	1=bis zu einer Woche; 2=bis zu zwei Wochen; 3=bis zu drei Wochen; 4=bis zu vier Wochen; 5=bis zu fünf Wochen; 6=bis zu sechs Wochen; 7=mehr als 6 Wochen	
	Haushaltshilfe Umfang	4.10 Wie viele Stunden in der Woche nutzen Sie eine Haushaltshilfe (Kochen, Putzen, Einkaufe)?	1=bis zu einer Stunde; 2=mehr als eine Stunde; 3=mehr als zwei Stunden; 4=mehr als drei Stunden; 5=mehr als vier Stunden	
	Betreuungsdienste Umfang	4.11 Wie viele Stunden in der Woche nutzen Sie einen Betreuungsdienst?	1=bis zu einer Stunde; 2=mehr als eine Stunde; 3=mehr als zwei Stunden; 4=mehr als drei Stunden; 5=mehr als vier Stunden	

	**Nutzung weiterer Unterstützungsangebote**			
	Beratung genutzt	6.1 Haben Sie sich schon einmal zu Pflegefragen beraten lassen?	1=Ja; 2=Nein	V (bei signifikantem Ergebnis
	**Wohnraumanpassende Maßnahmen**			
	Wohnraumanpassung	5.2 Haben Sie wegen der Pflege im Haus/ in der Wohnung Veränderungen oder Umbauarbeiten vorgenommen?	1=Ja; 2=Nein	Chi2 nach Pearson; Cramer V (bei signifikantem Ergebnis >0,05)
	**Einschätzung der Pflegesituation**			
	Einschätzung Pflegesituation	9.1 Wie schätzen Sie Ihre Pflegesituation insgesamt ein? Die häusliche Pflege ist...	1=sehr gut zu bewältigen; 2=noch zu bewältigen; 3=nur unter Schwierigkeiten zu bewältigen; 4=eigentlich gar nicht mehr zu bewältigen	Rangkorrelation nach Spearman (einseitig/gerichtet)
