# Supplementary material for: Homecare arrangements: examining the use of support services in relation to socioeconomic status
Source: Bundesgesundheitsblatt Gesundheitsforschung Gesundheitsschutz. 2023 Mar 20;66(5):540–9. [Article in German] doi: 10.1007/s00103-023-03684-6 (PMC10163119; doi:10.1007/s00103-023-03684-6)
Supplement: Supplementary file 2 [file 103_2023_3684_MOESM2_ESM.pdf]

Onlinematerial 2
Englert et al. (2023): Häusliche Pflegearrangements: Untersuchung der Inanspruchnahme von Unterstützungsleistungen im Zusammenhang mit dem sozioökonomischen Status
-Stichprobenbeschreibung-

Pflegebedürftige Personen nach SGB XI ab 55 Jahre, n=3.871		Häufgkeit n	Angabe in %
**Personenbezogene Merkmale der pflegebedürftigen Person**			
Alter (Pflichtmerkmal; n=3.871)	91 Jahre und älter	66	1,7
	86 bis 90 Jahre	104	2,7
	81 bis 85 Jahre	347	9
	76 bis 80 Jahre	383	9,9
	71 bis 75 Jahre	526	13,6
	66 bis 70 Jahre	604	15,6
	61 bis 65 Jahre	855	22,1
	55 bis 60 Jahre	986	25,5
Pflegegrad (Pflichtmerkmal; n=3.871)	Pflegegrad 1	596	15,4
	Pflegegrad 2	1.737	44,9
	Pflegegrad 3	1.121	29,0
	Pflegegrad 4	335	8,7
	Pflegegrad 5	82	2,1
Geschlecht (n=3.216)	männlich	1.602	49,8
	weiblich	1.607	50,0
	divers	7	0,2
**Sozioökonomische Merkmale der pflegebedürftigen Person**			
Haushaltseinkommen zusammengefasst (n= 3.433)	weniger als 1.000 €	400	11,7
	1.000 € bis 1.999 €	1.377	40,1
	2.000 € bis 2.999 €	985	28,7
	3.000 € bis 3.999 €	399	11,6
	mehr als 4.000 €	272	7,9
Bildungsabschluss zusammengefasst (n=3.150)	Hauptschulabschluss	907	28,8
	Realschulabschluss	918	29,1
	Fachhochschulreife/Abitur	778	24,7
	Hochschulabschluss/Promotion	547	17,4
**Merkmale des Pflegearrangements**			
Nutzung von Unterstützungsleistungen (n=3.610)	Pflegegeld	2.942	76,0
	Ambulanter Pflegedienst	881	22,8
	Tages-/Nachtpflege	83	2,1
	Verhinderungspflege	946	24,4
	Kurzzeitpflege	181	4,7
	Entlastungsbetrag	839	21,7
	Haushaltshilfe	1.339	34,6
	Betreuungsdienst	153	4,0
	24-Stunden-Pflege	44	1,1
	Keine Leistungen	219	5,7
Umfang genutzter Unterstützungsleistungen	Ambulanter Pflegedienst (in Minuten pro Tag; n=690)		
	1 bis 15 Minuten	180	26,1
	16 bis 30 Minuten	254	36,8
	31 bis 60 Minuten	144	20,9
	61 bis 90 Minuten	52	7,5
	91 bis 120 Minuten	28	4,1
	mehr als 120 Minuten	32	4,6
	Tages-/Nachtpflege (in Stunden pro Woche; n=72		
	1 bis 8 Stunden	23	31,9
	9 bis 16 Stunden	17	23,6
	17 bis 24 Stunden	9	12,5
	25 bis 32 Stunden	5	6,9
	33 bis 40 Stunden	2	2,8
	mehr als 40 Stunden	16	22,2
	Verhinderungs- und Ersatzpflege (Stunden wöchentlich in den letzten 12 Monaten; n=201)		
	1 bis 24 Stunden	141	70,1
	25 bis 48 Stunden	18	9
	49 bis 72 Stunden	10	5
	73 bis 96 Stunden	6	3
	97 bis 120 Stunden	9	4,5
	121 bis 144 Stunden	5	2,5
	144 bis 168 Stunden	12	6

Umfang genutzter Unterstützungsleistungen	Verhinderungs- und Ersatzpflege (Wochen in den letzten 12 Monaten; n=402)		
	bis 1 Woche	51	12,7
	mehr als 1 Woche bis 2 Wochen	52	12,9
	mehr als 2 Wochen bis 3 Wochen	75	18,7
	mehr als 3 Wochen bis 6 Wochen	145	36,1
	mehr als 6 Wochen bis 12 Wochen	30	7,5
	mehr als 12 Wochen	49	12,2
	Kurzzeitpflege (in Wochen im letzten Jahr; n=99		
	bis zu einer Woche	13	13,1
	bis zu zwei Wochen	23	23,2
	bis zu drei Wochen	28	28,3
	bis zu vier Wochen	16	16,2
	bis zu fünf Wochen	4	4
	bis zu sechs Wochen	2	2
	mehr als sechs Wochen	13	13,1
	Haushaltshilfe (in Stunden pro Woche; n=1.269)		
	bis zu einer Stunde	264	20,8
	mehr als eine Stunde	327	25,8
	mehr als zwei Stunden	277	21,8
	mehr als drei Stunden	152	12,0
	mehr als vier Stunden	249	19,6
	Betreuungsdienst (in Stunden pro Woche; n=113)		
	bis zu einer Stunde	21	18,6
	mehr als eine Stunde	30	26,5
	mehr als zwei Stunden	19	16,8
	mehr als drei Stunden	18	15,9
	mehr als vier Stunden	25	22,1
Wohnraumanpassende Maßnahmen (n=3.466)	Ja	2.032	58,6
	Nein	1.434	41,4
Nutzung von Beratung (n=3.370)	Ja	2.398	71,2
	Nein	972	28,8
Informelles Pflegenetz	Vorhandensein einer Pflegeperson (n=3.194)		
	Ja	2.749	86,1
	Nein	445	13,9
	Wohnsituation (n=3.038)		
	Mit der Hauptpflegeperson in einer Wohnung/einem Haus.	2.181	71,8
	Ohne die Hauptpflegeperson in einer Wohnung/einem Haus.	857	28,2
	Umfang Pflegeleistung Hauptpflegeperson (n=2.892)		
	unter 5 Stunden	400	13,8
	5 bis unter 10 Stunden	524	18,1
	10 bis unter 20 Stunden	691	23,9
	20 bis unter 30 Stunden	522	18
	30 bis unter 40 Stunden	305	10,5
	40 Stunden und mehr	450	15,6
	Unterstützung durch weitere Pflegepersonen (n=2.630)		
	Ja	1.425	54,2
	Nein	1.205	45,8
	Anzahl der Pflegepersonen (n=1.379)		
	eine Person	535	38,8
	zwei Personen	522	37,9
	drei Personen	201	14,6
	mehr als drei Personen	121	8,8
**Einschätzung des Pflegearrangements durch die pflegebedürftige Person**			
Einschätzung der Pflegesituation (n=3.377)	sehr gut zu bewältigen	903	26,7
	noch zu bewältigen	1.819	53,9
	nur unter Schwierigkeiten zu bewältigen	603	17,9
	eigentlich gar nicht mehr zu bewältigen	52	1,5

Pflegepersonen von pflegebedürftigen Personen nach SGB XI ab 55 Jahre, n=17.990		Häufgkeit n	Angabe in %
**Personenbezogene Merkmale der Pflegeperson und der pflegebedürftigen Person**			
Alter der Pflegeperson (n=13.867)	86 bis 95 Jahre	72	0,5
	76 bis 85 Jahre	778	5,6
	66 bis 75 Jahre	2.683	19,3
	56 bis 65 Jahre	7.302	52,7
	46 bis 55 Jahre	2.512	18,1
	36 bis 45 Jahre	381	2,7
	26 bis 35 Jahre	122	0,9
	16 bis 25 Jahre	17	0,1
Geschlecht der Pflegeperson (n=13.970)	männlich	4.158	29,8
	weiblich	9.802	70,2
	divers	10	0,1
Beziehung zur pflegebedürftigen Person (n=17.939)	mein Ehepartner/mein Lebensgefährte	4.224	23,5
	mein Vater/meine Mutter	10.265	57,2
	mein Schwiegervater meine Schwiegermutter	1.819	10,1
	mein Bruder/meine Schwester	229	1,3
	mit mir anderweitig verwandt (z.B. mein Onkel/ meine Tante)	659	3,7
	ein Freund oder ein Nachbar von mir	413	2,3
	Sonstige	330	1,8
Befragte Person ist Hauptpflegeperson (17.490)	Ja	13.119	75,0
	Nein	4.371	25,0
Wohnsituation (n=17.384)	Ohne die pflegebedürftige Person in einer Wohnung/einem Haus	9.270	53,3
	Mit der pflegebedürftigen Person in einer Wohnung/einem Haus	8.114	46,7
Alter der pflegebedürftigen Person (Pflichtmerkmal, n=17.990)	55 bis 60 Jahre	948	5,3
	61 bis 65 Jahre	962	5,3
	66 bis 70 Jahre	1163	6,5
	71 bis 75 Jahre	1434	8
	76 bis 80 Jahre	2558	14,2
	81 bis 85 Jahre	4465	24,8
	86 bis 90 Jahre	3974	22,1
	91 bis 95 Jahre	2058	11,4
	96 bis 100 Jahre	428	2,4
Pflegegrad der pflegebedürftigen Person (Pflichtmerkmal, n=17.990)	Pflegegrad 1	954	5,3
	Pflegegrad 2	4.865	27,0
	Pflegegrad 3	6.390	35,5
	Pflegegrad 4	3.774	21,0
	Pflegegrad 5	2.007	11,2
Geschlecht der pflegebedürftigen Person (n=17.892)	männlich	6.424	35,9
	weiblich	11.452	64,0
	divers	16	0,1
**Sozioökonomische Merkmale der Pflegeperson**			
Haushaltseinkommen (n=11.991)	weniger als 1.000 €	950	7,9
	1.000 € bis 1.999 €	3.605	30,1
	2.000 € bis 2.999 €	4.043	33,7
	3.000 € bis 3.999 €	1.940	16,2
	mehr als 4.000 €	1.453	12,1
Bildungsabschluss (n=13.477)	Hauptschulabschluss	3.065	22,7
	Realschulabschluss	4.714	35,0
	Fachhochschulreife/Abitur	3.449	25,6
	Hochschulabschluss/Promotion	2.249	16,7
**Merkmale des Pflegearrangements der pflegebedürftigen Person**			
Nutzung von Unterstützungsleistungen (n=16.031)	Pflegegeld	13.370	83,4
	Ambulanter Pflegedienst	7.007	43,7
	Tages-/Nachtpflege	1.283	8,0
	Verhinderungspflege	4.189	26,1
	Kurzzeitpflege	2.251	14,0
	Entlastungsbetrag	2.924	18,2
	Haushaltshilfe	4.024	25,1
	Betreuungsdienst	1.160	7,2
	24-Stunden-Pflege	1.044	6,5
	Keine Leistungen	732	4,6

Umfang genutzter Unterstützungsleistungen	Ambulanter Pflegedienst (in Minuten pro Tag; n=6.406)		
	1 bis 15 Minuten	1.490	23,3
	16 bis 30 Minuten	2.593	40,5
	31 bis 60 Minuten	1.562	24,4
	61 bis 90 Minuten	411	6,4
	91 bis 120 Minuten	150	2,3
	mehr als 120 Minuten	200	3,1
	Tages-/Nachtpflege (in Stunden pro Woche; n=1.198)		
	1 bis 8 Stunden	326	27,2
	9 bis 16 Stunden	283	23,6
	17 bis 24 Stunden	211	17,6
	25 bis 32 Stunden	108	9
	33 bis 40 Stunden	99	8,3
	mehr als 40 Stunden	171	14,3
	Verhinderungs- und Ersatzpflege (Stunden wöchentlich in den letzten 12 Monaten; n=934)		
	1 bis 24 Stunden	665	71,2
	25 bis 48 Stunden	110	11,8
	49 bis 72 Stunden	49	5,2
	73 bis 96 Stunden	29	3,1
	97 bis 120 Stunden	33	3,5
	121 bis 144 Stunden	18	1,9
	144 bis 168 Stunden	30	3,2
	Verhinderungs- und Ersatzpflege (Wochen in den letzten 12 Monaten; n=1.632)		
	bis 1 Woche	238	14,6
	mehr als 1 Woche bis 2 Wochen	353	21,6
	mehr als 2 Wochen bis 3 Wochen	377	23,1
	mehr als 3 Wochen bis 6 Wochen	474	29
	mehr als 6 Wochen bis 12 Wochen	75	4,6
	mehr als 12 Wochen	115	7
	Kurzzeitpflege (in Wochen im letzten Jahr; n=1.751)		
	bis zu einer Woche	216	12,3
	bis zu zwei Wochen	481	27,5
	bis zu drei Wochen	451	25,8
	bis zu vier Wochen	329	18,8
	bis zu fünf Wochen	56	3,2
	bis zu sechs Wochen	125	7,1
	mehr als sechs Wochen	93	5,3
	Haushaltshilfe (in Stunden pro Woche; n=3.795)		
	bis zu einer Stunde	906	23,9
	mehr als eine Stunde	1.111	29,3
	mehr als zwei Stunden	794	20,9
	mehr als drei Stunden	405	10,7
	mehr als vier Stunden	579	15,3
	Betreuungsdienst (in Stunden pro Woche; n=931)		
	bis zu einer Stunde	145	15,6
	mehr als eine Stunde	204	21,9
	mehr als zwei Stunden	180	19,3
	mehr als drei Stunden	119	12,8
	mehr als vier Stunden	283	30,4
Wohnraumanpassende Maßnahmen (n=14.772)	Ja	8.740	57,2
	Nein	6.546	42,8
Nutzung von Beratung (n=14.772)	Ja	10.962	74,2
	Nein	3.810	25,8
Teilnahme an Pflegekurs (n=14.781)	Ja	2.493	16,9
	Nein	12.288	83,1
Informelles Pflegenetz	Umfang der wöchentlichen Pflegeleistung (n=16.468)		
	unter 5 Stunden	1.045	6,3
	5 bis unter 10 Stunden	3.124	19,0
	10 bis unter 20 Stunden	4.564	27,7
	20 bis unter 30 Stunden	3037	18,4
	30 bis unter 40 Stunden	1687	10,2
	40 Stunden und mehr	3011	18,3

Informelles Pflegenetz	Unterstützung durch weitere Pflegepersonen (n=17.537)		
	Ja	10.541	60,1
	Nein	6.996	39,9
	Anzahl weiterer Pflegepersonen (n=10.402)		
	eine Person	4.802	46,2
	zwei Personen	3.463	33,3
	drei Personen	1.383	13,3
	mehr als drei Personen	754	7,2
**Einschätzung des Pflegearrangements durch die Pflegeperson**			
Einschätzung der Pflegesituation (n=13.800)	sehr gut zu bewältigen	1.518	11,0
	noch zu bewältigen	7.273	52,7
	nur unter Schwierigkeiten zu bewältigen	4.248	30,8
	eigentlich gar nicht mehr zu bewältigen	761	5,5
