# Supplementary material for: Homecare arrangements: examining the use of support services in relation to socioeconomic status
Source: Bundesgesundheitsblatt Gesundheitsforschung Gesundheitsschutz. 2023 Mar 20;66(5):540–9. [Article in German] doi: 10.1007/s00103-023-03684-6 (PMC10163119; doi:10.1007/s00103-023-03684-6)
Supplement: Supplementary file 3 [file 103_2023_3684_MOESM3_ESM.pdf]

Onlinematerial 3

Englert et al. (2023): Häusliche Pflegearrangements: Untersuchung der Inanspruchnahme von Unterstützungsleistungen im Zusammenhang mit dem sozioökonomischen Status

-Ergebnisse aller durchgeführten statistischen Analysen-

Farblegende:

- signifikant, ohne Effekt (p >0.05, V/rs < 0.1)
- signifikant, schwacher Effekt (p < 0.05, V/rs = 0.1)
- signifikant, moderater Effekt (p < 0.05, V/rs = 0.3)
- signifikant, starker Effekt (p < 0.05, V/rs = 0.5)

Stichprobe: Pflegebedürftige Personen nach SGB XI ab 55 Jahre

	Ergebnis Zusammenhangsanalyse Unabhängige Variable (Chi2 nach Pearson, Cramer's V)									
**Abhängige Variable**	**Haushaltseinkommen**					**Bildung**				
	**Chi2**	**df**	**p**	**V**	**n**	**Chi2**	**df**	**p**	**V**	**n**
**Nutzung von Unterstützungsleistungen**										
Pflegegeld (ab PG 2!)	25,537	4	0,000	0,097	2.738	8,831	3	0,032	0,060	2.481
Ambulanter Pflegedienst			Ø Sig.		3.241			Ø Sig.		2.944
Tages-/Nachtpflege			Ø Sig.		3.241			Ø Sig.		2.944
Verhinderungspflege			Ø Sig.		3.241			Ø Sig.		2.944
Kurzzeitpflege			Ø Sig.		3.241			Ø Sig.		2.944
Entlastungsbetrag			Ø Sig.		3.241			Ø Sig.		2.944
Haushaltshilfe			Ø Sig.		3.241			Ø Sig.		2.944
Betreuungsdienst			Ø Sig.		3.241			Ø Sig.		2.944
24-Stunden-Pflege	15,669	4	0,003	0,070	3.241			Ø Sig.		2.944
**Nutzung von Beratungsangeboten**										
Beratung genutzt	13,709	4	0,008	0,067	3.048			Ø Sig.		2.746
**Wohnraumanpassende Maßnahmen**										
Wohnraumanpassung	**87,892**	**4**	**0,000**	**0,168**	**3.121**			Ø Sig.		2.820

	Ergebnis Zusammenhangsanalyse Unabhängige Variable (Spearman-Rangkorrelation)					
**Abhängige Variable**	**HHE**			**Bildung**		
	**rs**	**p**	n	**rs**	**p**	**n**
**Umfang genutzter Unterstützungsleistungen**						
Pflegedienst Umfang	0,081	0,021	630		Ø Sig.	560
Tages-/Nachtpflege Umfang		Ø Sig.	69		Ø Sig.	63
Verhinderungspflege Umfang Stunden/Woche		Ø Sig.	189		Ø Sig.	156
Verhinderungspflege Umfang Wochen/Jahr		Ø Sig.	362		Ø Sig.	335
Kurzzeitpflege Umfang		Ø Sig.	88		Ø Sig.	83
Haushaltshilfe Umfang	0,072	0,007	1.136	-0,054	0,043	1.012
Betreuungsdienste Umfang		Ø Sig.	104		Ø Sig.	89
**Einschätzung der Pflegesituation**						
Einschätzung Pflegesituation	**-0,142**	**0,000**	**3.045**		Ø Sig.	2.755

Stichprobe: Pflegebedürftige Personen nach SGB XI ab 55 Jahre nach Pflegerad<br>n=596 (PG1); 1.737 (PG2); 1.121 (PG3); 335 (PG4); 82 (PG5)						
**Ergebnis Zusammenhangsanalyse Unabhängige Variable (Chi2 nach Pearson, Cramer's V)**						
**Abhängige Variable**	**Pflegegrad**	**Haushaltseinkommen**				
		**Chi2**	**df**	**p**	**V**	**n**
**Nutzung von Unterstützungsleistungen**						
Pflegegeld	2	**20,637**	**4**	**0,000**	**0,118**	**1.486**
	3			Ø Sig.		917
	4			Ø Sig.		265
	5			Ø Sig.		70
Ambulanter Pflegedienst	1					503
	2	14,152	4	0,007	0,098	1.486
	3			Ø Sig.		917
	4			Ø Sig.		265
	5			Ø Sig.		70
Verhinderungspflege	1					503
	2	9,842	4	0,043	0,081	1.486
	3			Ø Sig.		917
	4			Ø Sig.		265
	5			Ø Sig.		70
Entlastungsbetrag	1			Ø Sig.		503
	2			Ø Sig.		1.486
	3			Ø Sig.		917
	4			Ø Sig.		265
	5			Ø Sig.		70
Haushaltshilfe	1			Ø Sig.		503
	2			Ø Sig.		1.486
	3			Ø Sig.		917
	4			Ø Sig.		265
	5			Ø Sig.		70
**Nutzung von Beratungsangeboten**						
Beratung genutzt	1			Ø Sig.		477
	2			Ø Sig.		1.391
	3			Ø Sig.		866
	4			Ø Sig.		249
	5			Ø Sig.		65
**Wohnraumanpassende Maßnahmen**						
Wohnraumanpassung	1	**13,907**	**4**	**0,008**	**0,170**	**484**
	2	**23,891**	**4**	**0,000**	**0,129**	**1.429**
	3	**31,713**	**4**	**0,000**	**0,189**	**884**
	4	**13,26**	**4**	**0,010**	**0,228**	**256**
	5			Ø Sig.		68

Stichprobe: Pflegebedürftige Personen nach SGB XI ab 55 Jahre nach Pflegerad<br>n=596 (PG1); 1.737 (PG2); 1.121 (PG3); 335 (PG4); 82 (PG5)				
**Ergebnis Zusammenhangsanalyse Unabhängige Variable (Spearman-Rangkorrelation)**				
**Abhängige Variable**	**Pflegegrad**	**Haushaltseinkommen**		
		**rs**	**p**	**n**
**Umfang genutzter Unterstützungsleistungen**				
Pflegedienst Umfang	1		Ø Sig.	49
	2		Ø Sig.	249
	3		Ø Sig.	215
	4		Ø Sig.	88
	5		Ø Sig.	29
Haushaltshilfe Umfang	1		Ø Sig.	185
	2	0,085	0,028	509
	3		Ø Sig.	320
	4		Ø Sig.	96
	5		Ø Sig.	26
**Einschätzung der Pflegesituation**				
Einschätzung Pflegesituation	1	**-0,198**	**0,000**	**474**
	2	**-0,151**	**0,000**	**1.394**
	3	**-0,127**	**0,000**	**864**
	4		Ø Sig.	249
	5		Ø Sig.	64

Stichprobe: Pflegepersonen von pflegebedürftigen Personen nach SGB XI ab 55 Jahre (n=17.990)										
**Ergebnis Zusammenhangsanalyse Unabhängige Variable (Chi2 nach Pearson)**										
**Abhängige Variable**	**Haushaltseinkommen**					**Bildung**				
	**Chi2**	**df**	**p**	**V**	**n**	**Chi2**	**df**	**p**	**V**	**n**
**Nutzung von Unterstützungsleistungen**										
Pflegegeld			Ø Sig.		11.930	31,511	3	0,000	0,048	13.400
Ambulanter Pflegedienst	47,502	4	0,000	0,063	11.930	52,306	3	0,000	0,062	13.400
Tages_Nachtpflege	12,031	4	0,017	0,032	11.930	14,659	3	0,002	0,033	13.400
Verhinderungspflege			Ø Sig.		11.930	8,367	3	0,039	0,025	13.400
Kurzzeitpflege			Ø Sig.		11.930			Ø Sig.		13.400
Entlastungsbetrag	21,701	4	0,000	0,043	11.930	80,680	3	0,000	0,078	13.400
Haushaltshilfe	43,053	4	0,000	0,060	11.930	69,479	3	0,000	0,072	13.400
Betreuungsdienst			Ø Sig.		11.930	21,547	3	0,000	0,040	13.400
Vier_Zwanzig_Stunden_Pflege	76,126	4	0,000	0,080	11.930	75,956	3	0,000	0,075	13.400
**Nutzung weiterer Unterstützungsangebote**										
Beratung_genutzt	17,921	4	0,001	0,039	11.513	112,041	3	0,000	0,093	12.889
**Wohnraumanpassende Maßnahmen**										
Wohnraumanpassung	22,542	4	0,000	0,044	11.674	20,392	3	0,000	0,039	13.101

**Ergebnis Zusammenhangsanalyse Unabhängige Variable (Spearman-Rangkorrelation)**						
**Abhängige Variable**	**HHE**			**Bildung**		
	**rs**	**p**	**n**	**rs**	**p**	**n**
**Umfang genutzter Unterstützungsleistungen**						
Pflegedienst_Umfang	0,060	0,000	4.764	0,060	0,000	5.373
Tagespflege/Nachtpflege_Umfang		Ø Sig.	931		Ø Sig.	1.036
Verhinderungspflege_Umfang_Stunden_Woche		Ø Sig.	738		Ø Sig.	818
Verhinderungspflege_Umfang_Wochen_Jahr		Ø Sig.	1.284	0,082	0,002	1.412
Kurzzeitpflege_Umfang		Ø Sig.	1.338		Ø Sig.	1.512
Haushaltshilfe_Umfang	0,014	0,000	2.942	**0,134**	**0,000**	**3.265**
Betreuungsdienste_Umfang		Ø Sig.	717		Ø Sig.	810
**Einschätzung der Pflegesituation**						
Einschätzung_Pflegesituation	-0,031	0,001	11.527	0,092	0,000	12.907

Stichprobe: Pflegepersonen von pflegebedürftigen Personen nach SGB XI ab 55 Jahre, die sich als Hauptpflegeperson angeben (n=13.119)										
**Ergebnis Zusammenhangsanalyse Unabhängige Variable (Chi2 nach Pearson, Cramer's V)**										
**Abhängige Variable**	**Haushaltseinkommen**					**Bildung**				
	**Chi2**	**df**	**p**	**V**	**n**	**Chi2**	**df**	**p**	**V**	**n**
**Nutzung von Unterstützungsleistungen**										
Pflegegeld			Ø Sig.		9.116	20,907	3	0,000	0,045	10.173
Ambulanter Pflegedienst	39,733	4	0,000	0,066	9.116	41,300	3	0,000	0,064	10.173
Tages_Nachtpflege			Ø Sig.		9.116	11,430	3	0,010	0,034	10.173
Verhinderungspflege			Ø Sig.		9.116	8,467	3	0,037	0,029	10.173
Kurzzeitpflege			Ø Sig.		9.116			Ø Sig.		10.173
Entlastungsbetrag	21,506	4	0,000	0,049	9.116	76,391	3	0,000	0,087	10.173
Haushaltshilfe	43,123	4	0,000	0,069	9.116	64,454	3	0,000	0,080	10.173
Betreuungsdienst			Ø Sig.		9.116	11,971	3	0,007	0,034	10.173
Vier_Zwanzig_-Stunden_Pflege	51,022	4	0,000	0,075	9.116	54,725	3	0,000	0,073	10.173
**Nutzung weiterer Unterstützungsangebote**										
Beratung_genutzt	17,577	4	0,001	0,045	8.845	79,865	3	0,000	0,090	9.837
**Wohnraumanpassende Maßnahmen**										
Wohnraumanpassung	20,646	4	0,000	0,048	8.944	9,361	3	0,025	0,031	9.969

**Ergebnis Zusammenhangsanalyse Unabhängige Variable (Spearman-Rangkorrelation)**						
**Abhängige Variable**	**HHE**			**Bildung**		
	**rs**	**p**	**n**	**rs**	**p**	**n**
**Umfang genutzter Unterstützungsleistungen**						
Pflegedienst_Umfang	0,057	0,001	3.311	0,051	0,001	3.705
Tagespflege/Nachtpflege_Umfang		Ø Sig.	724		Ø Sig.	796
Verhinderungspflege_Umfang_Stunden_Woche		Ø Sig.	626		Ø Sig.	688
Verhinderungspflege_Umfang_Wochen_Jahr		Ø Sig.	1079	-0,087	0,001	1.177
Kurzzeitpflege_Umfang		Ø Sig.	1038		Ø Sig.	
Haushaltshilfe_Umfang	**0,146**	**0,000**	**2.243**	**0,121**	**0,000**	**2.475**
Betreuungsdienste_Umfang		Ø Sig.	551		Ø Sig.	617
**Einschätzung der Pflegesituation**						
Einschätzung_Pflegesituation	-0,038	0,000	8.867	0,095	0,000	9863

Stichprobe: Pflegepersonen von pflegebedürftigen Personen nach SGB XI ab 55 Jahre, differenziert nach Beziehungsstatus zur pflegebedürftigen Person; n= 4.224 (Partner); 10.265 (Mutter/Vater)											
**Ergebnis Zusammenhangsanalyse Unabhängige Variable (Chi2 nach Pearson, Cramer's V)**											
**Abhängige Variable**	**Beziehung zur pflegebedürftigen Person**	**Haushaltseinkommen**					**Bildung**				
		**Chi2**	**df**	**p**	**V**	**n**	**Chi2**	**df**	**p**	**V**	**n**
**Nutzung von Unterstützungsleistungen**											
Pflegegeld	(Ehe-)Partner*in			Ø Sig.		3.126			Ø Sig.		3.419
	Mutter/ Vater			Ø Sig.		6.675	23,119	3	0,000	0,055	7.596
Ambulanter Pflegedienst	(Ehe-)Partner*in			Ø Sig.		3.126			Ø Sig.		3.419
	Mutter/ Vater	43,913	4	0	0,081	6.675	32,817	3	0,000	0,066	7.596
Tages_Nachtpflege	(Ehe-)Partner*in			Ø Sig.		3.126			Ø Sig.		3.419
	Mutter/ Vater			Ø Sig.		6.675	7,856	3	0,049	0,032	7.596
Verhinderungspflege	(Ehe-)Partner*in			Ø Sig.		3.126			Ø Sig.		3.419
	Mutter/ Vater	11,778	4	0,019	0,042	6.675	7,899	3	0,048	0,032	7.596
Kurzzeitpflege	(Ehe-)Partner*in			Ø Sig.		3.126			Ø Sig.		3.419
	Mutter/ Vater	15,839	4	0,003	0,049	6.675			Ø Sig.		7.596
Entlastungsbetrag	(Ehe-)Partner*in	20,920	4	0,000	0,082	3.126	30,583	3	0,000	0,095	3.419
	Mutter/ Vater			Ø Sig.		6.675	57,595	3	0,000	0,087	7.596
Haushaltshilfe	(Ehe-)Partner*in	29,444	4	0,000	0,097	3.126	13,718	3	0,003	0,063	3.419
	Mutter/ Vater	23,029	4	0,000	0,059	6.675	52,891	3	0,000	0,083	7.596
Betreuungsdienst	(Ehe-)Partner*in	16,368	4	0,003	0,072	3.126			Ø Sig.		3.419
	Mutter/ Vater			Ø Sig.		6.675	9,383	3	0,025	0,035	7.596
Vier_Zwanzig_-Stunden_Pflege	(Ehe-)Partner*in	14,930	4	0,005	0,069	3.126	8,860	3	0,031	0,051	3.419
	Mutter/ Vater	53,281	4	0,000	0,089	6.675	51,791	3,000	0,000	0,083	7.596
**Nutzung weiterer Unterstützungsangebote**											
Beratung_genutzt	(Ehe-)Partner*in			Ø Sig.		3.034	**48,009**	**3**	**0,000**	**0,121**	**3.305**
	Mutter/ Vater			Ø Sig.		6.429	**68,465**	**3**	**0,000**	**0,097**	**7.301**
Pflegekurs	(Ehe-)Partner*in			Ø Sig.		3.066			Ø Sig.		3.334
	Mutter/ Vater	21,124	4	0,000	0,057	6.437			Ø Sig.		7.323
**Wohnraumanpassende Maßnahmen**											
Wohnraumanpassung	(Ehe-)Partner*in	26,831	4	0,000	0,094	3060	17,275	3	0,001	0,072	3341
	Mutter/ Vater			Ø Sig.		6.538	16,686	3	0,001	0,047	7.431
**Informelles Pflegenetz**											
Hauptpflegeperson	(Ehe-)Partner*in			Ø Sig.		3.112	14,374	3	0,002	0,065	3.401
	Mutter/ Vater	54,048	4	0,000	0,091	6.542	9,944	3	0,019	0,037	7.454
Weitere_Pflegepersonen	(Ehe-)Partner*in			Ø Sig.		3.069	14,083	3	0,003	0,065	3.351
	Mutter/ Vater	**119,379**	**4**	**0,000**	**0,134**	**6.607**	15,461	3	0,001	0,045	7.518
Wohnsituation	(Ehe-)Partner*in			Ø Sig.		3.087			Ø Sig.		3.373
	Mutter/ Vater	**82,764**	**4**	**0,000**	**0,112**	**6.565**	59,655	3	0,000	0,089	7.470

Stichprobe: Pflegepersonen von pflegebedürftigen Personen nach SGB XI ab 55 Jahre, differenziert nach Beziehungsstatus zur pflegebedürftigen Person; n= 4.224 (Partner); 10.265 (Mutter/Vater)							
**Ergebnis Zusammenhangsanalyse Unabhängige Variable (Spearman-Rangkorrelation)**							
**Abhängige Variable**		**HHE**			**Bildung**		
		**rs**	**p**	**n**	**rs**	**p**	**n**
**Umfang genutzter Unterstützungsleistungen**							
Pflegedienst_Umfang	(Ehe-)Partner*in		Ø Sig.	766	0,072	0,019	833
	Mutter/ Vater	0,060	0,001	3.042	0,060	0,000	3.460
Tagespflege/Nachtpflege_Umfang	(Ehe-)Partner*in	**0,128**	**0,023**	**241**		Ø Sig.	260
	Mutter/ Vater		Ø Sig.	522		Ø Sig.	587
Verhinderungspflege_Umfang_Stunden_Woche	(Ehe-)Partner*in		Ø Sig.	245		Ø Sig.	267
	Mutter/ Vater		Ø Sig.	404		Ø Sig.	447
Verhinderungspflege_Umfang_Wochen_Jahr	(Ehe-)Partner*in		Ø Sig.	328	-0,105	0,025	343
	Mutter/ Vater		Ø Sig.	708	-0,060	0,046	799
Kurzzeitpflege_Umfang	(Ehe-)Partner*in		Ø Sig.	252		Ø Sig.	272
	Mutter/ Vater		Ø Sig.	832		Ø Sig.	
Haushaltshilfe_Umfang	(Ehe-)Partner*in	**0,177**	**0,000**	**781**	0,057	0,049	842
	Mutter/ Vater	**0,126**	**0,000**	**1.684**	**0,170**	**0,000**	**1.891**
Betreuungsdienste_Umfang	(Ehe-)Partner*in		Ø Sig.	181		Ø Sig.	198
	Mutter/ Vater		Ø Sig.	402		Ø Sig.	461
**Einschätzung der Pflegesituation**							
Einschätzung_Pflegesituation	(Ehe-)Partner*in	-0,36	0,025	3.057	0,071	0,000	3338
	Mutter/ Vater	-0,038	0,001	6419	0,092	0,000	7277
